# Exogenous Nitric Oxide Induced Early Mineralization in Rat Bone Marrow Mesenchymal Stem Cells via Activation of Alkaline Phosphatase

**DOI:** 10.29252/.23.2.142

**Published:** 2019-03

**Authors:** Mohammad Hussein Abnosi, Sadieeh Pari

**Affiliations:** Department of Biology, Faculty of Sciences, Markazi, Iran

**Keywords:** Alkaline phosphatase, Mesenchymal stem cells, Nitroprusside, Osteoblasts

## Abstract

**Background:**

Since the low concentration and short-time treatment with sodium nitroprusside (SNP), a nitric oxide (NO)–donor, cause no harm to rat bone marrow mesenchymal stem cells (MSCs), we studied the impact of SNP on MSCs differentiation.

**Methods:**

MSCs were treated with 100 and 1000 µM of SNP for 1 hour in every 48 hours and after 5, 10, 15, and 21 days in osteogenic media. The viability and the level of mineralization were determined using MTT assay and alizarin red staining, respectively. Morphology of the cells was studied using fluorescent dye. Concentration of calcium and the activity of alanine transaminase (ALT), aspartate transaminase (AST), lactate dehydrogenase (LDH), and alkaline phosphatase (ALP) were evaluated by commercial kits.

**Results:**

SNP with the concentration of 1000 µM significantly reduced viability from day 5 to day 20, but 100 µM did not affect the viability until the day 15. The low concentration of SNP increased matrix deposition from day 10 and reached almost its maximum (4.40 ± 2.4) at the day 15. Also, increasing the activity of ALP (419 ± 2.2), due to low concentration of SNP, started at day 10 and continued till the day 20, while LDH (2026 ± 11) and AST (25.6 ± 0.4) elevations were observed from day 5 onwards. In case of ALT, we observed a significant decrease (36%) from day 5 till day 20.

**Conclusion:**

Based on our findings, low concentrations of SNP might be useful in the promotion of bone repair.

## INTRODUCTION

Nitric oxide (NO), in the cells, is produced by NO synthase (NOS) and has cell signaling activity^[^^1^^]^. In 1772, Joseph Priestley discovered NO and called it ‘nitrous air’, but till 1987 this molecule was considered as an air pollutant. Later, it was shown to be formed in the animal body by NOS and was determined to act as a vasodilator, which is used to treat various cardiovascular diseases^[^^2^^]^. NOS has three isoenzymes called as neuronal form (type 1; nNOS), endothelial form (type 3; eNOS) and inducible form (type 2; iNOS)^[^^3^^]^, which produce NO in different cells. 

NO has the short half-life of 5-10 seconds; therefore, its action is confined to the adjacent area. When entering the cell, NO targets the guanylate cyclase^[^^4^^]^ and increases the formation of cAMP that finally governs its action. In bone-related cells, such as mesenchymal stem cells (MSCs), osteoblasts, and osteocytes, two isoforms of NO synthase (eNOS and iNOS) are expressed, whereas in osteoclasts, only nNOS is produced. In osteoblast, the NO acts as a double agent that regulates cell survival as well as cell death. At low concentration, NO is associated with cell proliferation, while high concentrations of NO cause apoptosis, which can be related to different pathological conditions^[^^5^^,^^6^^]^.

As quoted before^[^^5^^,^^6^^]^, NO, depending on its concentration, modulates the activity of both osteoblasts and osteoclasts *in vitro*. Besides, it has an anabolic effect on bone tissue and stimulates mineralization. Production of the large amounts of NO by the cells inhibits osteoblast proliferation and causes bone resorption mediated by osteoclasts^[^^7^^]^. Irrespective of production in the cells, NO may be generated via compounds such as nitroglycerine and sodium nitroprusside (SNP). SNP is an arterial and a venous vasodilator used in clinical practice to lower blood pressure. It is a water-soluble sodium salt having Fe^2+^, NO, and five cyanide anions in its structure^[^^8^^]^. In biological systems, SNP releases NO via non-enzymatic and enzymatic processes^[^^9^^]^. In the body, SNP functions as a pro-drug, reacting with sulfhydryl groups on erythrocytes, albumin, and other proteins to release NO, which immediately decreases the blood pressure in clinical situations^[^^8^^]^. Repair of fractured bone and wound healing after bone surgery requires differentiation of bone marrow MSCs to osteoblasts^[^^10^^]^. MSCs possess two fundamental characteristics: the ability to proliferate and the capacity to differentiate^[^^11^^,^^12^^]^. 

Considerable *in vitro *and animal studies suggest that MSCs have the potential of rapid bone regeneration that can differentiate to osteoblasts in bone tissues. Some other investigations have studied the effect of SNP on bone marrow MSCs. Chu *et al.*^[^^13^^]^ have found that the treatment of adult mouse bone marrow multipotent progenitor cells with 500 to 2000 µM of SNP for 48 hours can reduce cell proliferation significantly. Felka *et al.*^[^^10^^]^ have shown that the concentration of 10 and 25 μM of SNP activates respiratory activity to some extent. However, application of SNP at 100-500 μM reduces the respiratory activity of MSC to approximately 80%, whereas 1 mM or higher decreases the respiratory activity of MSC to 30% or 20%. Recently, we have demonstrated that the treatment of the MSCs with 100 µM of SNP for 1 hour causes no change in the morphology and viability of these cells after 24 hours of incubation. However, incubation of MSCs for 7 and 14 days reduced the numbers of colony. In addition, biochemical analysis of the cells displayed an increase in anaerobic metabolism coupled with the reduction of alkaline phosphatase (ALP) activity, while calcium concentration remained unchanged^[^^14^^]^. 

To the best of our knowledge, no work has been published to discuss the possible effect of SNP, as an NO-releasing agent, on the differentiation property of MSCs to osteoblasts. It is well documented that the osteoblasts generate NO enzymatically, showing the key role in osteoblasts activity. Therefore, in this research work, we tried to figure out the effect of exogenous NO on MSCs osteogenic differentiation.

## MATERIALS AND METHODS

**Isolation and expansion of bone marrow mesenchymal stem cells**

In this experimental study, Wistar rats (6-8 weeks old) were purchased from Pasteur Institute of Iran (Tehran) and kept in the animal house of Arak University (Markazi Province) under standard condition of light and food. The animals were sacrificed by excessive chloroform inhalation, and then their tibia as well as femur were removed and cleaned from adherent soft tissue. Next, the two ends of the bones were cut off, and bone marrow was flash out using 2-ml DMEM (Gibco, Germany) supplemented with 15% FBS and 1 % penicillin-streptomycin (10 ml/L; both from Gibco, Germany). Bone marrow content was centrifuged at 2500 rpm for 5 minute and re-suspended in 5-ml DMEM, then plated in culture flasks and incubated at 37 °C in an atmosphere of 5% CO_2_. After 24 hours, the old medium was replaced by fresh medium. The medium exchange was repeated two times a week till the bottom of the flask was covered with the cells (i.e. till confluency). The cells were trepsinized (trypsin-EDTA, Gibco, Germany) and passed to another culture flask as the first passage, and then the cultures were expanded through two additional subcultures at which the cells were used for further investigation^[^^15^^]^. All the procedures were approved by the Experimental Animal Ethics Committee of the Arak University (No. P/97/2S3016). 

**Osteogenic induction**

Mineralization was induced in a confluent monolayer of cells by the addition of DMEM containing 15% (v/v) FBS, 1 % (v/v) streptomycin-penicillin (10 ml/L), and osteogenic supplements (1 mM sodium glycerophosphate, 50 μg/ml L-ascorbate, and 10^-8^ M dexamethasone). All the chemicals were purchased from Sigma-Aldrich Company (USA) unless mentioned elsewhere. The culture flasks were then incubated at 37 °C with 5% CO_2_, and their medium was changed every three days for 21 days^[^^15^^]^. 

**Exposure to sodium nitroprusside**

SNP (Merck, Germany) was used to make 100 and 1000 µM concentration of SNP in the culture media, and the pH of the solution was adjusted to 7.3**.** The treatment with SNP was carried out for one hour in every 48 hours, and the cells were cultured in media without SNP for 5, 10, 15 and 21 days in the presence of osteogenic supplements. The same procedure was carried out with the control group where no SNP treatment was done.

**Cell viability assays**

The viability test on control and treated cells was carried out using MTT (4,5dimethylthiazol-2-yl)-2,5-diphenyltetrazolium bromide) assay. In live cells, after 4 h of incubation, mitochondrial succinate dehydro-genase converts yellow color tetrazolium into violet crystal of formazan. At room temperature, 100 μL of DMSO was added to each well of the plate, and formazan crystals were extracted following 30 minute of incubation. The solution was transferred into another well, and the absorbance was measured at 505 nm using an automated microplate reader (SCO Diagnostic, Germany)^[^^15^^]^. 

**Analysis of morphological changes**

Following SNP treatment, the cells were cultured in an osteogenic medium for 5, 10, 15, and 21 days. To study the nuclear morphology, the cells were stained for 5 minutes in a solution of 1 µg/ml of Hoechst 33342 in the dark at room temperature and then examined under an inverted fluorescence microscope (Olympus, IX70, Japan). The diameter of the cells was measured in μm with the help of Motic Image software (Micro optical group company version 1.2). Hoechst is a fluorescent dye that penetrates the cells through the intact plasma membrane and stains the DNA where the changes in nuclear morphology such as chromatin condensation and fragmentation can be investigated^[16]^. In addition, using acridine orange, as a fluorescent dye, the morphology of the cell cytoplasm was investigated. After staining, the cells were washed twice with PBS, examined and photographed using an inverted fluorescence microscope (Olympus, IX70) equipped with a camera.

**Detection and quantification of mineralization**

Treated and control cells were cultured in 6-well plates for 5, 10, 15, and 21 days, then were washed with PBS and fixed in 10% (v/v) formaldehyde at room temperature for 15 minute. During the staining procedure, the cells were washed twice with excess of dH_2_O, and subsequently, 1 mL of 40 mM alizarin red solution (pH 4.1) was added per well. The plates were incubated at room temperature for 20 minute with gentle shaking, and excess of dye was discarded. The plates were washed four times with dH_2_O, and stained cells were investigated under a light-inverted microscope and photographed. To quantify the level of absorbed alizarin red, 800 μL of 10% acetic acid (v/v) was added to each well, and the plate was incubated at room temperature for 30 minute with gentle shaking. Afterwards, the loosely attached cells were scraped from the plate with a cell scraper and transferred to a 1.5-mL micro-centrifuge tube. After vortexing for 30 second, the slurry was overlaid with 500 μL mineral oil, heated at 85 °C for 10 minute and kept on ice for 5 minute. The slurry was centrifuged at 12870 g for 15 minutes, 500 μL of the supernatant was transferred to a new microcentrifuge tube, and 200 μL of 10% ammonium hydroxide (v/v) was added to neutralize the acid. An aliquot of the supernatant (100 μL) was read in triplicate at 405 nm in a microplate reader (SCO Diagnostic, Germany) and quantified against standard graph. To prepare alizarin red standards graph, working alizarin red solution (40 mM) was diluted 20 times with a mixture of 5:2 of 10% acetic acid and 10% ammonium to give a concentration of 2000 μM. Different standard solutions of 2000 to 31.3 μM were prepared, and the absorbancess were recorded at 450 nm using a microplate reader. The concentration of the unknown samples was calculated using the linear formula Y = 0.099X + 0.101 with R^2 ^= 0.997, where Y is the absorbance, and X is the concentration (mM) of alizarin red^[^^16^^]^.

**Preparation of cell extract**

Control and osteogenic-treated cells were washed with Tris-HCl buffer. The loosely attached cells were scraped off the plate with the cell scraper and grinded in liquid nitrogen, and the cell content was then extracted with Tris-HCl buffer, followed by centrifugation at 12870 g for 10 minute. The total protein content of each sample was determined by Lowry method^[^^17^^]^ using bovine serum albumin as standard. Standard graph was plotted, and the concentration of the unknown protein samples was calculated using the linear formula Y=0.0021X + 0.0271 with R^2^ = 0.994, where Y is the absorbance, and X is the concentration (μg) of the protein in each sample. 

**Determination of alkaline phosphatase activity**

ALP activity of protein lysate was determined by a commercially available kit (Parsazmon, Iran). In brief, to determine the activity of enzyme, 20 µl of the sample was mixed with 1000 µl of the 1^st^ reagent (diethanolamine [1.0 mol/l; pH 9.8] and magnesium chloride [0.5 mmol/l]). Subsequently, the tubes were shaken for 10 second and incubated for 1 minute and then the 2^nd^ reagent (p-nitophenylphosphage [10 mmol/l]) was added. Finally, the absorbance was measured after 1 minute at 405 nm using a spectrophotometer (T80 + PG instrument Ltd., England).

**Determination of transaminases and lactate dehydrogenaseactivity**

Alanine transaminase (regent 1: 100 mmol/l of Tris [pH 7.5], 500 mmol/l of L-alanine, ≥1200 U/l of lactate dehydrogenase and reagent 2: 2-oxogutarate [15 mmol/l] and NADH [0.18 mmol/l]), aspartate transaminase (reagent 1: Tris [80 mmol/l; pH 7.8], L-aspartate [240 mmol/l], malat dehydrogenase [≥600 U/l] and lactate dehydrogenase (≥600 U/l) and reagent 2: 2-oxoglutarate [12 mmol/l] and NADH [0.18 mmol/l]), and lactate dehydrogenase (regent 1: phosphate buffer [50 mmol/l ; pH 7.5], pyruvate [0.6 mmol/l] and reagent 2: Good’s buffer (pH 9.6), NADH 0.18 mmol/l) activities were determined (in protein lysate of control and treated samples based on an equal amount of protein in each one) according to a commercial kit instruction (Parsazmon, Iran). In brief, for determination of transaminase and lactate dehydrogenase activity, 100 and 10 µl of the sample, respectively were mixed with 1000 µl of the 1^st^ reagent for 1 minutes. Then 250 µl of 2^nd^ reagent was added to the tubes, and absorbance was measured for 1 minute at 340 nm using a spectrophotometer (T80, PG Instrument Ltd., England). 

**Fig. 1:**
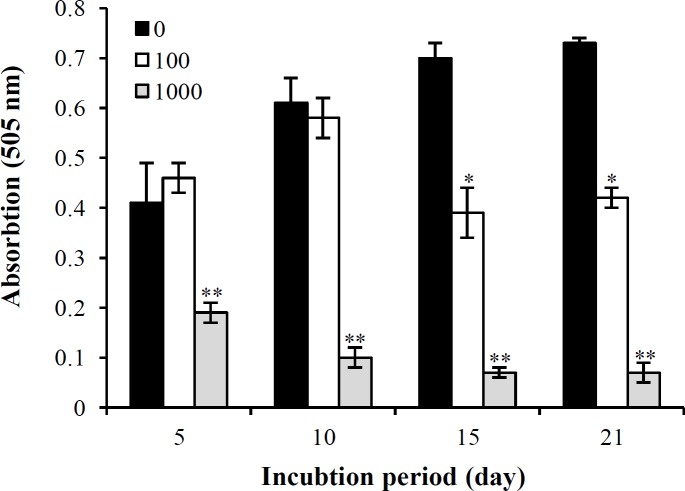
The cell viability of BMSCs after 5, 10, 15, and 21 days of treatment with 0, 100, and 1000 µM of SNP in osteogenic media, based on MTT assay. Values are means ± SD. The asterisks (^*^) and (^**^) represent the level of significant in each day compared to the control (*p* < 0.05 and *p* < 0.001, respectively; ANOVA, Tukey's test)

**Calcium concentration**

Both control and treated cells were washed twice with PBS and then were incubated for 24 hours with 50 μl of 0.5 N HCl to dissolve the calcium content. The amount of calcium was determined using the commercial kit (Parsazmon, Iran). In detail, to determine the concentration of calcium, 10 µl of the sample was mixed with 1000 µl of the reagent (phosphate buffer [pH 7.5; 50 mmol/l], 8-hydroxyquinoline-5-sulfonic acid [5 mol/l], and arsenazoIII [120 µmol/l]). The tubes were then shaken, and absorbance of the developed color was measured at 575 nm using a spectrophotometer (T80, PG Instrument Ltd., England). Using different concentrations of calcium chloride, a standard curve was prepared, and the concentration of unknown samples was calculated using the linear formula as follows: Y = 0.0763X-0.0039 with R^2 ^= 0.998, where Y is the absorbance, and X is the concentration (mg/dl) of calcium. 

**Statistical analysis**

Statistical evaluation of the data was performed using one-way analysis of variance (ANOVA) and Tukey's test, with the help of SPSS (version 16). Results were shown as mean ± SD, and *p* < 0.05 was accepted statistically as the minimum level of statistical significance. 

## RESULTS

**Effect of** **SNP on cell viability **

MTT assay showed that the 100 µM of SNP did not change the viability at 5 and 10 days of treatment, but at 15 and 21 days, it significantly decreased (*p *< 0.05) the bone marrow MSC viability under osteogenic differentiation. On the other hand, the 1000 µM of SNP caused a highly significant (*p* < 0.001) reduction in viability on days 5, 10, 15, and 21, as compared with the control (Fig. 1). 

**Morphological changes in differentiated MSCs**

**Fig. 2:**
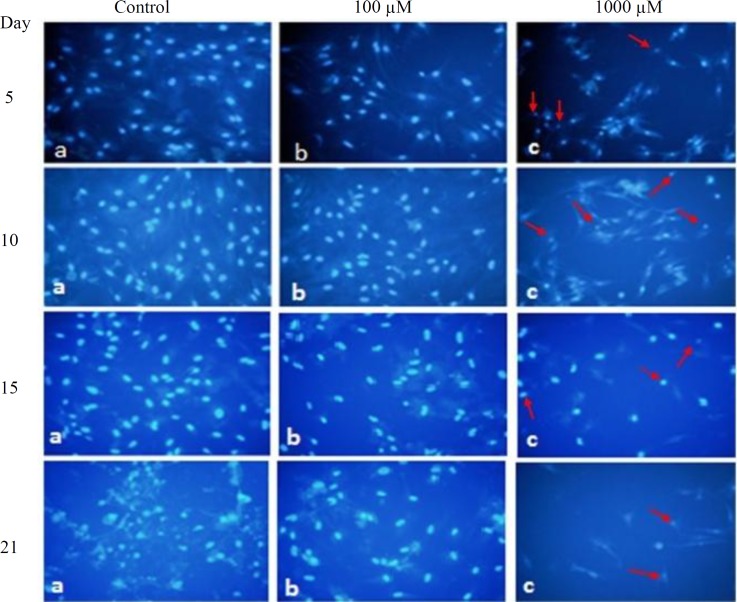
Fluorescent micrograph images of BMSCs stained with Hoechst, after 5, 10, 15, and 21days of incubation in osteogenic media treated with 0, 100 and 1000 µM of SNP. Nuclear condensation and deformation (arrows) of cells were observed after treatment with 1000 µM of SNP (magnification 200 ).

**Fig. 3:**
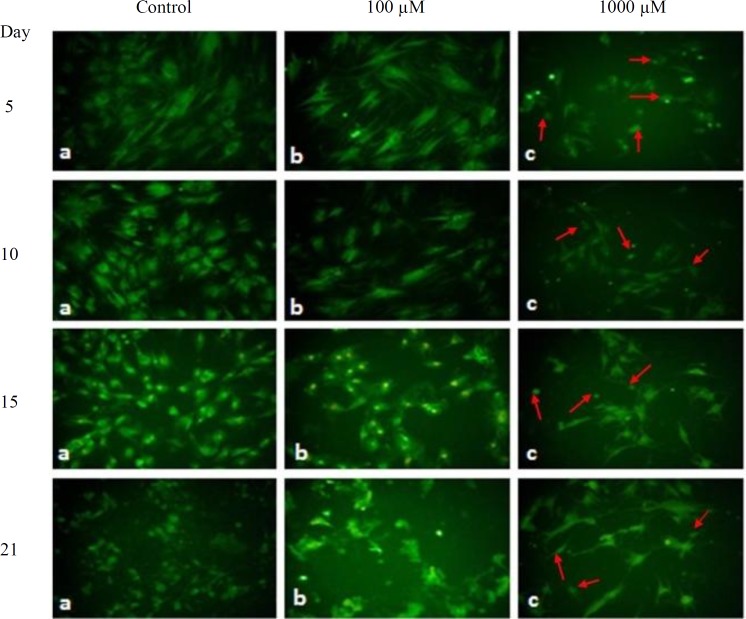
Fluorescent micrograph images of BMSCs stained with acridine orange, after 5, 10, 15, and 21 days of incubation in osteogenic media treated with 0, 100 and 1000 µM of SNP. Shrinkage and complete disappearance of cytoplasm in some cells (arrows) were observed after treatment with 1000 µM of SNP (magnification 200 )

Morphological study of the nuclei in differentiated MSCs after 5, 10, 15 and 21 days of treatment with 1000 µM of SNP revealed chromatin condensation and breakage (Fig. 1), as well as significant reduction (*p* < 0.05) in nuclei diameter (Table 1). It could also be noticed that SNP at 1000 µM concentration caused remarkable changes in the morphology of cytoplasm such as shrinkage and complete disappearance of cytoplasm in some cells (Table 2 and Fig. 2). The concentration of 100 µM of SNP after 5, 10, 15, and 21 days induced no significant changes in the morphology of cytoplasm and nuclei (Figs. 2 and 3) as well as nuclei diameter and cytoplasm area (Tables 1 and 2) compared to the control. On the other hand, with passing time from 5 to 21 days of osteogenic incubation, the control showed morphological changes from mesenchymal lineage to osteoblast-differentiated cells. As the size of the nuclei and cytoplasm reduced, the cells became round, and the nuclei was centric. 

**Table 1:** Mean nuclear diameter of mesenchymal stem cells after 5, 10, 15, and 21 days of treatment with different doses of SNP

**Doses (µM)**	**Nucleus diameter** ** (µm)**			
**Days**	**5**	**10**	**15**	**21**
0	11.70^a ^± 0.42	11.03^a ^± 0.17	9.73^a ^± 0.23	6.58^a ^± 0.27
100	11.78^a ^± 0.25	10.64^a ^± 0.18	9.62^a ^± 0.23	7.02^a ^± 0.33
1000	6.53^b ^± 0.13	6.03^b ^± 0.16	6.02^b ^± 0.18	3.7^b ^± 0.19

Values are means ± SD. Means with the same letter do not differ significantly from each other in a column (ANOVA, Tukey's test, *p* > 0.05).

**Table 2:** Mean cytoplasm area of mesenchymal stem cells after 5, 10, 15, and 21 days of treatment with different doses of SNP

**Doses (µM)**	**Cytoplasm area** ** (um** ^2^ **)**			
**Days**	**5**	**10**	**15**	**21**
0	2768.92^a ^± 99.3	1903.17^a ^± 84.4	1951.27^a ^± 81.9	1015.37^a ^± 40.6
100	2735.63^a ^± 11.38	1896.08^a ^± 8.59	1900.78^a ^± 58.5	998.57^a ^± 68.4
1000	1573.73^b ^± 11.58	1581.27^b ^± 13.9	1617.58^b ^± 84.3	928.85^b ^± 54.0

Values are means ± SD. Means with the same letter do not differ significantly from each other in a column (ANOVA, Tukey's test, *p* > 0.05).

**Mineralization analysis based on alizarin red and calcium content**

**Fig. 4:**
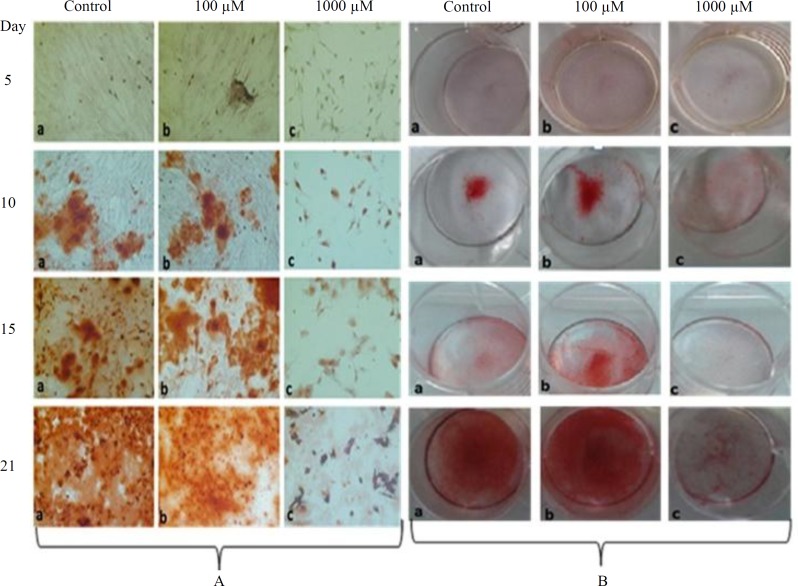
Osteogenic confirmation. (A) Microscopic images (200 ) of the cell after alizarin red staining; (B) Camera photograph from plates after alizarin red staining

Data analysis showed that the mineralization of MSCs in the control group started at day 10, but the presence of 100 µM concentration of SNP significantly increased (*p* < 0.05) the mineralization based on alizarin red and calcium deposition (Tables 3 and 4). Microscopic picture and camera photograph of alizarin red confirmed the qualitative analysis when compared to the control (Fig. 4). In the control group, mineralization in the absence of SNP mainly started at day 10 and reached its maximum at day 21; however, the presence of 100 µM SNP made a highly significant difference (*p* < 0.001) on day 15, as compared to the control, but at day 21, it did not show any difference (Tables 3 and 4; Fig. 4A and 4B). The high concentration of SNP (1000 µM) caused a significant reduction (*p* < 0.05) in differentiation ability at 5, 10, 15 and 21 days of treatment when compared to the control cells, as well as lower concentration treated cells (100 µM of SNP).

**Metabolic activity of the cells**

**Fig. 5:**
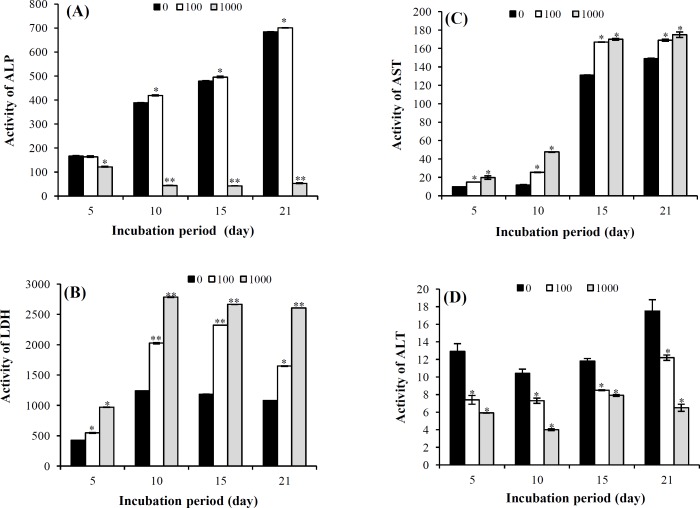
Mean activity of ALP (A), LDH (B), AST (C), and ALT (D) in the cells at osteogenic media after 5, 10, 15 and 21days of incubation and treatment with 0, 100, and 1000 µM of SNP. Values are means ± SD. The asterisks (^*^) and (^**^) represent the level of significant in each day compared to the control at *p* < 0.05 and *p* < 0.001, respectively (ANOVA, Tukey's test)

In the control group, ALP activity increased at day 10 and reached its maximum at day 21. Although the activity of the same enzyme in the group of the cells treated with 100 µM of SNP started from day 10, but it was more significant (*p* < 0.05) than the control group. Also, at days 15 and 21, it continued with the same significant increase in comparison with the control. Treatment of the cells with 1000 µM of SNP resulted in a high significant reduction (*p* < 0.001) of ALP activity at the days 5, 10, 15, and 21 (Fig. 5A). The LDH activity in the control group showed an increasing trend from day 5 to the day 21 under the influence of differentiation medium. The increase of LDH activity in the control group was more obvious in day 10 and 15 compared to day 5 and 21. The treatment of the cells with SNP caused a 

**Table 3:** Effect of different concentrations of SNP on mineralization based on alizarin red after 5, 10, 15, and 21 days of incubation in osteogenic media

**Doses (µM) **	**5**	**10**	**15**	**21**
**Days**				
0	1.51^a ^± 0.15	2.39^a ^± 0.14	2.69^a ^± 0.8	4.71^a ^± 0.15
100	1.43^a ^± 0.57	2.98^b ^± 0.15	4.40^b ^± 0.24	4.59^a ^± 0.19
1000	1.08^a ^± 0.59	1.00^b ^± 0.44	1.55^c ^± 0.50	1.48^b ^± 0.11

Values are means ± SD. Means with the same letter do not differ significantly from each other in a column (ANOVA, Tukey's test, *p* > 0.05).

**Table 4:** Effect of SNP on calcium (mg/dl) concentration after treatment with 0, 100, and 1000 µM of SNP

**Doses (µM)**	**5**	**10**	**15**	**21**
**Days**				
0	1.97^a^ ± 0.5	26.04^a^ ± 0.39	29.81^a^ ± 1.15	35.06^a^ ± 0.48
100	2.16^a^ ± 0.17	28.20^b^ ± 0.79	31.98^b^ ± 1.90	34.96^a^ ± 0.48
1000	0.77^b^ ± 0.57	14.45^b^ ± 1.99	17.73^c^ ± 1.24	20.82^a^ ± 1.05

Values are means ± SD. Means with the same letter do not differ significantly from each other in a column (ANOVA, Tukey's test, *p* > 0.05).

significant increase in the activity of LDH in all the treatment periods, but on days 10 and 15, it was highly significant (*p* < 0.001) for both 100 and 1000 µM. Increase in the LDH activity was concentration-dependent as we observed in each period (Fig. 5B). In case of AST activity, a very similar observation compared to the activity of LDH could be made. In the control group, the activity increased with the elevation of time. SNP treatment also caused a significant increase in AST when compared to the control group (Fig. 5C). In case of ALT activity, SNP caused a significant dose-dependent reduction (*p* < 0.05), when compared to the control group at all the treatment periods (Fig. 5D). 

## DISCUSSION

Studies have shown that the NO is able to arrest cell cycle that consequently inhibits cell proliferation in most cases^[^^18^^,^^19^^]^. In our study, we showed that treatment (1 hour in every 48 hours) with 100 µM of SNP did not have any toxicity effect within 10 days, but it was toxic and reduced cell viability in longer time. Floryszak-Wieczorek *et al.*^[^^20^^]^ have estimated that the half-life of SNP is 12 h in a culture system, but in addition to NO production, SNP also releases cyanide and iron^[^^21^^]^. Since SNP with the concentration of 100 μM generates only 1.2 nM of NO^[^^22^^]^ and vanishes very quickly, the presence of cyanide in the culture medium could be the reason for viability reduction observed via cell toxicity from day 5 with respect to 1000 µM treatment. Evidence has shown that the cyanide produced by SNP is concentration-dependent^[^^23^^]^; thus, our observation with higher concentration might be due to more production of cyanide that inhibits the cell respiration via mitochondrial dysfunction^[^^24^^]^. 

In this study, the differentiation medium caused morphological changes in the control cells, which were marked by reduction in the cytoplasm area and nuclei diameter. The changes in cytoplasm started at day 10, but nuclear diameter reduction started at day 15 and continued till day 21. SNP at 100 µM concentration did not bring about differences in the morphology and its developmental phenomena when compared with the control group. However, higher concentration (1000 µM) caused the shrinkage of cytoplasm and nuclear condensation, which all together are considered to be a sign of apoptosis^[^^25^^]^. It is well documented that the high concentration, but not low concentration, of NO induces apoptosis^[^^26^^-^^28^^]^, which at physiological level is considered as a signaling molecule^[^^29^^]^. Researchers have also mentioned many drastic effects such as viability reduction, morphological changes, and formation of cell debris when periodontal ligament fibroblasts is treated with different concentrations (1 to 4 mM) of SNP for 16 hours^[^^30^^]^, which is in agreement with our findings at high concentration. Irrespective of cell toxicity and mortality caused by the high concentration of SNP, which mainly might be due to cyanide toxicity, Huitema *et al.*^[^^31^^]^ have found that the treatment of ATDCS cell line with low concentration (100 μM) of SNP for 24 hours causes the inhibition of cell mineralization. In addition to cyanide, a toxic compound, that can inhibit cytochrome *C *oxidase in the respiratory chain^[^^23^^]^, inorganic iron derived from SNP has been indicated to be able to inhibit the effect of mineralization^[^^31^^]^. As a matter of fact, SNP can generate reactive oxygen species, including hydroxyl, which can induce lipid peroxidation and cytotoxicity^[^^32^^]^. Unlike Huitema et al.'s^[^^31^^]^ study, we observed that 100 µM concentration of SNP did not show toxicity and mineralization inhibition. In the present study, the cells were pretreated with SNP for a short time (only 1 hour in every 48 hour), and then the osteogenic process was carried out; therefore, neither the cyanide and iron toxicity can be considered as a causative matter nor the concentration of SNP is enough to create the problem of oxidative stress. 

One of the key findings of the present study is the promotion of the MSCs differentiation to osteoblasts, which appears to show the important role of NO at lower concentration. In the control group, under the influence of osteogenic medium, mineralization took place at day 10 and reached its maximum at day 21. Nonetheless, cell treatment with low concentration (100 µM) of SNP caused the cells deposit more calcium in the matrix at day 10 and reach the high level at day 15. It seems that the low concentration of SNP released enough NO to activate the genes, which bring about the early differentiation. Previous studies have shown that NO is involved in endothelial progenitor cell growth and differentiation via gene activation^[^^33^^,^^34^^]^. Oct-4 expression in mouse embryonic stem cells is widely considered as a hallmark of cell pluripotency and critical to the regulation of embryonic differentiation^[^^35^^,^^36^^]^. Chu *et al.*^[^^13^^]^ have found that NO increases Oct-4 expressions in bone marrow stem cells, suggesting that NO may be important to maintain their pluripotency. Therefore, we can conclude that the genes involved in MSCs differentiation to osteoblasts such as ALP may up-regulate with SNP. 

ALP is a critical enzyme involved in bone matrix mineralization. Its expression starts at the early stage of cell differentiation and continues till the final stage of MSCs differentiation to osteoblasts^[^^37^^]^. In the present study, the activity of ALP, under the influence of low concentration of SNP, was significantly more than that of the control group at days 10, 15, and 21. As we quoted before, the activation of genes such as ALP might be the reason for mineralization activation. Thus, we can deduce that although the high concentration of SNP was toxic and reduced the activity of ALP, low concentration of SNP significantly increased the activity of this enzyme from day 10. Mechanistically, ALP releases the phosphate and causes the calcium (Ca^++^) to enter the cell and being deposited along with phosphate to form hydroxyapatite crystal^[^^37^^]^; this observation has also been observed in our study. 

Mineralization is essential for normal skeletal development, which is mainly accomplished through the function of osteoblasts^[^^38^^]^. In differentiation process, the metabolic state of the cells changes, and the alteration in cell metabolism controls the cell development through its metabolites^[^^39^^]^. Differentiation of MSCs to osteoblasts caused the activity of LDH to be increased from day 10 in the control group, which indicates the shift of the metabolism from aerobic to anaerobic respiration. It is called Warburg effect that is characterized by the high rate of glycolysis and low mitochondrial oxidation of pyruvate despite high levels of O_2_ availability^[^^40^^]^. During differentiation, MSCs utilize both oxidative phosphorylation and glycolysis pathways^[^^41^^]^, where initially, total ATP levels and the ATP/ADP ratio are high, but it decreases linearly during differentiation. Considering that ATP is a potent allosteric inhibitor of PFK1, decrease in ATP may play a role in activating glycolysis at the osteocyte stage. On the other hand, when the NADH/NAD^+^ ratio is high to increase the rate of glycolysis, the NADH/NAD^+^ ratio should reduce. Therefore, the activation of LDH is required to consume NADH and produce NAD. Our data showed that in the control cells, when the mineralization starts (day 10), the Warburg effect is maximum compared to the days 15 and 21. This result shows that at the early stage of differentiation, more energy is required, and cell has to go through this metabolic change. However, the situation continues till the end of differentiation process in a milder manner where the activity of LDH reduces, when compared to the day 10. Treatment of the cells with SNP caused the activity of LDH to be increased more significantly compared to the control group, showing more energy production through glycolysis pathway. This outcome indicates that NO might be a regulator of mineralization via metabolic changes. In accordance with LDH activation, AST activity has increased probably to form more oxaloacetate^[^^42^^]^, which can be used in two ways: (1) activate the krebs cycle by further using pyruvate, and (2) make more pyruvate via pyruvate carboxylase activity^[^^43^^]^. In the current study, ALT activity confirmed the overactivation of LDH and AST, where SNP caused the inhibition of its activation to consume less pyruvate. Although treatment of the cells with high concentration of SNP (1000 µM) showed the activation of LDH and AST, the nature of this activation might be quite different. It seems that this concentration caused the impairment of mitochondrial respiratory mechanism and therefore, made the cells to be faced with poor energy production, which brings about cell mortality.

 In conclusion, despite the cell toxicity induced by the high concentration of SNP, our analysis showed that the SNP at low concentration not only does not show any adverse effect but also reduces the differentiation time of MSCs to osteoblasts and increases matrix mineralization via the activation of ALP activity and metabolic changes.
